# Basement membrane collagen IV deficiency promotes abdominal aortic aneurysm formation

**DOI:** 10.1038/s41598-021-92303-y

**Published:** 2021-06-18

**Authors:** L. B. Steffensen, J. Stubbe, J. S. Lindholt, H. C. Beck, M. Overgaard, M. Bloksgaard, F. Genovese, S. Holm Nielsen, M. L. T. Tha, S. K. Bang-Moeller, M. K. T. Hong Lin, J. H. Larsen, D. R. Hansen, G. T. Jones, M. J. Bown, M. A. Karsdal, L. M. Rasmussen

**Affiliations:** 1 Centre for Individualized Medicine in Artery Diseases, Odense University Hospital, J. B. Winsløws Vej 4, 5000 Odense C, Denmark; 2 Department of Molecular Medicine, University of Southern Denmark, Odense, Denmark; 3 Department of Clinical Biochemistry and Pharmacology, Odense University Hospital, Odense, Denmark; 4 Department of Cardiothoracic and Vascular Surgery, Odense University Hospital, Odense, Denmark; 5 Nordic Bioscience, Herlev, Denmark; 6 Biomedicine and Biotechnology, Technical University of Denmark, Lyngby, Denmark; 7 University of Otago, Dunedin, Otago, New Zealand; 8 Department of Cardiovascular Sciences and the NIHR Leicester Biomedical Research Centre, University of Leicester, Leicester, UK

**Keywords:** Aneurysm, Translational research

## Abstract

Abdominal aortic aneurysm (AAA) is a complex disease which is incompletely accounted for. Basement membrane (BM) Collagen IV (COL4A1/A2) is abundant in the artery wall, and several lines of evidence indicate a protective role of baseline COL4A1/A2 in AAA development. Using *Col4a1/a2* hemizygous knockout mice (*Col4a1/a2*^+*/−*^, 129Svj background) we show that partial Col4a1/a2 deficiency augmented AAA formation. Although unchallenged aortas were morphometrically and biomechanically unaffected by genotype, explorative proteomic analyses of aortas revealed a clear reduction in BM components and contractile vascular smooth muscle cell (VSMC) proteins, suggesting a central effect of the BM in maintaining VSMCs in the contractile phenotype. These findings were translated to human arteries by showing that COL4A1/A2 correlated to BM proteins and VSMC markers in non-lesioned internal mammary arteries obtained from coronary artery bypass procedures. Moreover, in human AAA tissue, MYH11 (VSMC marker) was depleted in areas of reduced COL4 as assessed by immunohistochemistry. Finally, circulating COL4A1 degradation fragments correlated with AAA progression in the largest Danish AAA cohort, suggesting COL4A1/A2 proteolysis to be an important feature of AAA formation. In sum, we identify COL4A1/A2 as a critical regulator of VSMC phenotype and a protective factor in AAA formation.

## Introduction

Abdominal aortic aneurysm (AAA) is a chronic expansion of the abdominal aorta diameter of ≥ 30 mm. The development of AAA is usually asymptomatic until sudden rupture of the aortic wall resulting in excessive blood loss into the abdominal cavity, which is associated with > 80% mortality. Approximately 1.5% of males over the age of 65 die from AAA rupture. Current management of the disease is limited to screening, watchful waiting and surgical intervention once the aortic diameter reaches 55 mm^1^.

The pathology of AAA involves vascular inflammation, proteolytic degradation of structural extracellular matrix (ECM), artery wall remodeling, vascular smooth muscle cell (VSMC) phenotype modulation and -apoptosis^2^. Aside from age and male sex, the strongest risk factors for AAA are smoking and a family history of the disease, while, hyperglycemia and diabetes mellitus are negatively associated with AAA^3,4^.

As for most tissues, arteries comprise two types of ECM: Interstitial matrix and basement membrane (BM). The artery BM is located at the base of the endothelial lining and throughout the medial layer, where it encapsulates VSMCs^5,6^. The principal component of the artery BM is Collagen IV hemi-trimers consisting of two ⍺1 (COL4A1) chains and one ⍺2 (COL4A2) chain. Unlike fibrillar collagens, Collagen IV hemi-trimers cross-link to form a meshwork, which along with laminins assemble into a scaffold for additional BM constituents to bind. As such, the artery BM serves as an adhesive substrate for vascular cells to anchor, and at the same time directly (by integrin binding) or indirectly (by regulating diffusion of macromolecules to and from cells) maintains cellular homeostasis^7^.

COL4A1 and COL4A2 are encoded by their homonymous genes, which are organized head-to-head on opposite strands and separated by a 126-bp bidirectional promoter on chromosome 13 (13q34)^7^. The locus harboring *COL4A1/A2* is a highly replicated susceptibility locus for coronary- and peripheral artery disease, respectively^8,9^, and the relevance of the BM in artery biology is evident from rare familial syndromes caused by missense mutations in *COL4A1*. Aneurysms of carotid and cerebral arteries are common features of these syndromes, collectively termed *hereditary angiopathy with nephropathy, aneurysms, and muscle cramps* (*HANAC* syndrome)^10,11^, underlining the importance of COL4A1/A2 integrity in the artery wall. Moreover, we previously reported that non-lesioned arteries from diabetic patients, which display relative protection from AAA development (of note, the relative contribution of hyperglycemia per se^4^ and metformin intake^12^ to AAA protection observed in diabetics remains elusive) have a distinct elevation in BM proteins lead by a 40% increase in COL4A1/A2—an observation in line with reports on increased of COL4A1/A2 in diabetic vascular organoids^13^ and with the well-known accumulation of BM in small vessels^14^.

Based on the above observations, we hypothesize that the level of COL4A1/A2 in arteries (as a consequence of genetic or environmental factors) is a critical determinant for AAA formation.

The objective of this study was to test the hypothesis that COL4A1/A2 is a protective factor in AAA disease combining a novel mouse model of Col4a1/a2 deficiency with proteomic analysis of non-lesioned artery and AAA tissue from human patients.

## Materials and methods

### Mouse procedures

Generation of *Col4a1/a2*^+*/−*^ mice (129/Svj) (a kind gift from Ernst Pöschl, University of East Anglia, Norwich, UK) have been described previously^15^. *Col4a1/a2*^+*/−*^ mice were crossed with *Col4a1/a2*^+*/*+^ littermates to generate *Col4a1/a2*^+*/−*^ and *Col4a1/a2*^+*/*+^ offspring at a 1:1 ratio for experimental procedures. Primers used for genotyping were 5′CATCGCCTTCTATCGCCTTCTTGACG, 5′CTGGCTCAGAGCGTCTTG, and 5′ACCACCTCTGAGTTTCTGGA resulting in 351 bp and 610 bp amplicons for WT and KO alleles, respectively. Mice used for general characterization (12 weeks of age) and wire myography experiments (9–11 weeks of age) were euthanized by CO_2_/O_2_ inhalation and tissues were either snap frozen in liquid nitrogen or immersed in 4% formaldehyde in phosphate-buffered saline (PBS) for 24 h and then transferred to PBS (0.05% sodium azide) at 4 °C. Exclusion criteria set before experiments were death, or euthanasia of mice showing pre-defined humane endpoints. No mice were excluded from the presented experiments except for three *Col4a1/a2*^+*/*+^ mice that died in the first two days after PPE surgery due to post-surgery related issues reducing the *Col4a1/a2*^+*/*+^ group to *n* = 14. No data were excluded from analysis. Mouse group allocation was random, concealed, and all experimental procedures and analyses were performed blinded to the observers. The study is reported in accordance with ARRIVE guidelines. All procedures were performed in accordance with protocols approved by the local authorities (the Danish Animal Experiments Inspectorate) and conform to the guidelines from Directive 2010/63/EU of the European Parliament on the protection of animals used for scientific purposes.

### PPE model of AAA

Male 13–14 week-old mice were anaesthetized (ketaminol 100 mg/kg, xylazine 10 mg/kg) and the baseline infrarenal aortic lumen diameter during systole was determined by ultrasonography using the LOGIQ e portable ultrasound imaging system (GE healthcare) with a 22 MHz central frequency probe (L10-22-RS, GE healthcare). Mice underwent laparotomy and the infrarenal aorta was isolated. The baseline infrarenal outer aortic diameter during systole was determined from video recordings captured using a Leica Camera attached to the microscope. Then the infrarenal aorta was occluded using silk sutures and aortotomy was introduced allowing infusion of porcine pancreatic elastase (PPE) (1.5 units/ml, Sigma Aldrich) via polyethylene catheter expanding the aorta to twice the baseline diameter for 5 min, followed by flushing of the aorta and abdomen cavity with physiological saline. The aortotomy was closed with a suture, blood flow was restored, and the laparotomy was closed. At day 7 and 14 post-surgery, the maximal infrarenal aortic lumen diameter during systole was determined by ultrasonography in mice anesthetized by isoflurane. At day 14, the infrarenal aorta was isolated in anaesthetized mice and a video of the aorta was recorded to determine the maximal infrarenal outer aortic diameter during systole. Mice were sacrificed, aortas were isolated and fixed in 4% formaldehyde overnight, then changed to PBS (0.05% sodium azide).

### Wire myography

Abdominal aortas were removed from mice euthanized by CO_2_/O_2_ inhalation, dissected free of perivascular adipose tissue, transferred to ice cold physiological salt solution (PSS, 115 mM NaCl, 25 mM NaHCO_3_, 2.5 mM K_2_HPO_4_, 1.2 mM MgSO_4_, 5.5 mM glucose, 10 mM HEPES, and 1.3 mM CaCl_2_) and mounted on 200 µm pins in a Danish Myo Technology multi wire myograph system (Danish Myo Technology, Søften, Denmark). After mounting, segments were allowed to rest at 37 °C for 30 min in calcium free PSS supplemented with 1 mM EGTA and 3 µM SNP. Chambers were continuously aerated with 5% CO_2_ in air. Then, segments were stretched in steps of 200 µm, every 3 min, to an internal diameter and wall tension corresponding to 150 mmHg (20 kPa) according to the Law of Laplace. Following, tension was reduced to 100 mmHg (13.3 kPa), buffer changed to PSS, and segments allowed to rest for 30 min before smooth muscle viability was tested by addition of 60 mM K^+^ or 10 µM phenylephrine, and endothelial cell viability by addition of increasing concentrations of acetylcholine. Finally, stretch was released to 50% and segments were fixed in 4% formaldehyde overnight, before embedding in paraffin. Segments were cut perpendicularly to the length of the aorta, and wall and media areas determined on Masson trichrome stained sections. Wall stress and strain was calculated, and β-values determined as reported previously^15^ on basis of wall thicknesses estimated from the morphometry. Method adapted from Mulvany et al.^16^.

### qPCR

RNA was extracted from snap frozen tissue using NORGEN Total RNA Purification Kit (37500, Norgen Biotek Corp.) and cDNA was synthesized by High-Capacity cDNA Reverse Transcription Kit (4368814, Thermo Fisher Scientific). qPCR was performed using SYBR™ Green PCR Master Mix (4364344, Thermo Fisher Scientific) and the ViiA7 Real-Time PCR system (Thermo Fisher Scientific). Primers used were: *Col4a1*: 5′GGCCCTTCATTAGCAGGTGT and 5′GTGAGGACCAACCGTTAGGG; *Col4a2*: 5′CCCGGATCTGTACAAGGGTG and 5′CGCCTTTTGAGATTACGCCG; *Gapdh*: 5′GGCTGCCCAGAACATCAT and 5′CGGACACATTGGGGGTAG. *Gapdh* was used as reference gene.

### Preparing human artery tissue for proteomics

Non-lesioned internal mammary arteries (IMAs) from coronary artery bypass graft (CABG) procedures were obtained from the Odense Artery Biobank. Immediately after tissue harvest, IMAs were systematically dissected and stored at − 80 °C until liquid chromatography tandem-mass spectrometry (LC–MS/MS)-analysis as previously described^5^. All donors had given written consent, and the study was performed in accordance with protocols approved by the local ethics committee (S-20100044) and conformed to the principles of the Declaration of Helsinki.

### Proteomics

Preparation of samples for mass spectrometry have been described previously^17^. Snap frozen unchallenged aortas or formalin-fixed AAA segments from mice were homogenized using a TissueLyser system (Qiagen) with stainless steel beads (Qiagen) in a lysis buffer [100 mM DTT, 5% sodium deoxycholate, 1% β-octylglucoside, 20 mM Tris, pH 8.8, supplemented with complete, Mini, EDTA-free Protease Inhibitor Cocktail Tablets and PhosSTOP (Roche)]. Proteins were then denatured (99 °C for 20 min, 80 °C for 100 min), alkylated (150 mM iodoacetamide for 30 min at room temperature), precipitated (− 20 °C acetone for 1 h), resolubilized (8 M urea for 30 min at room temperature), and trypsinized (0.5 µg/µl trypsin (Promega) in 50 mM ammonium bicarbonate, 1 M urea at 37 °C overnight). After acidification, tryptic peptides were purified on custom-made Poros R2/R3 (Thermo Scientific) micro-columns, dried, reconstituted, and finally resuspended in 2% acetonitrile (ACN), 0.1% formic acid. The concentration of tryptic peptides was determined (Pierce BCA Protein Assay Kit, Thermo Fisher Scientific) and normalized across samples.

**Figure 1:**
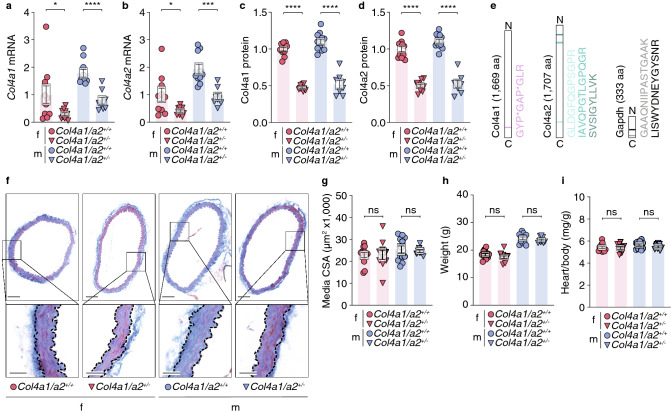
*Col4a1/a2* hemizygosity reduces aorta Col4a1/a2 content but does not affect morphometry of the aorta. (**a**, **b**) Aorta *Col4a1* (**a**) and *Col4a2* (**b**) mRNA levels relative to *Gapdh* measured by qPCR. (**c**, **d**) Aorta Col4a1 (**c**) and Col4a2 (**d**) protein levels relative to Gapdh measured by MRM LC–MS. For (**a**–**d**), data are normalized to the mean of the female *Col4a1/a2*^+*/*+^ group. (**e**) Peptides (and their location) of Col4a1, Col4a2 and Gapdh from the MRM LC–MS analysis used to obtain results in (**c**, **d**). P* = hydroxyproline, aa = amino acids. (**f**) Representative cross-sections of aortas stained by Masson trichrome. Scalebars (overview) = 100 µm; Scalebars (magnifications) = 25 µm. (**g**) Quantitation of aorta media cross-sectional area (analysis is based on Acta2-stained aorta sections exemplified in Supplementary Fig. 1). (**h**, **i**) Weight (**h**) and relative heart mass (**i**) of baseline *Col4a1/a2*^+*/−*^ mice and *Col4a1/a2*^+*/*+^ littermates. Data are presented as mean ± SEM. *p ≤ 0.05, ***p ≤ 0.001, ****p ≤ 0.0001, ns = non-significant. Unpaired t-test was used in a-d and g-i. Although both sexes are plotted, no intersex comparisons were performed. f = female, m = male. *n* per group = 8–12.

Targeted MRM analysis on tissue samples was adapted from previously described procedure^18^. Heavy isotope-labelled standard peptides (JPT Peptide Technologies) were added to each sample aiming at a 0.1–10 peak area ratio between each endogenous peptide assayed and its corresponding heavy peptide. Each sample (1 µg endogenous tryptic peptides) was run on an Easy-nLC II NanoLC system using a C18 trapping column (length 2 cm; internal diameter 100 µm) for desalting and a C18 analytical column (length 10 cm, internal diameter 75 µm) for peptide separation (Thermo Scientific). Peptides were eluted with a three-step 45 min gradient of 0.1% formic acid in ACN at a flow rate of 300 nl/min. Peptides were ionized using a Nanospray Flex ion source (Thermo Scientific) and analyzed on a TSQ Vantage triple quadruple mass spectrometer (Thermo Scientific) in MRM mode. MRM raw files were processed using Pinpoint 1.3 (Thermo Scientific). The peak area ratio between endogenous and heavy isotope labelled spiked peptide was used for data analysis. Peak area ratios for Col4a1 (target peptide: GYP*GAP*GLR, P* indicates hydroxyproline) and Col4a2 (target peptides: GLDGFQGPSGPR, IAVQPGTLGPQGR, SVSIGYLLVK) were normalized to Gapdh (target peptides: GAAQNIIPASTGAAK and LISWYDNEYGYSNR) within the same sample (Fig. 1e).

For explorative proteomics of mouse aortas, samples (4 µg tryptic peptides per sample) were randomly labelled with 10-plex tandem mass tags (TMT, Thermo Scientific) and combined into 2 × 3 samples, high-pH fractionated, and analyzed by nano-LC–MS/MS virtually as previously described^17^.

All Eclipse raw data files were processed and quantified using Proteome Discoverer version 2.4 (Thermo Scientific) as previously described^17^.

Proteomic analysis of human IMAs was based on formalin-fixed paraffin-embedded sections of IMAs obtained from CABG surgery. For this study, we analyzed the proteomic dataset of the 30 non-diabetic patients. Please refer to the original article for details on patients and proteomic analysis^5^.

### Histology and immunohistochemistry of mouse and human artery tissue

Fixed mouse tissue was cryoprotected in 25% sucrose in PBS (0.05% sodium azide) (24 h), 50% sucrose in PBS (0.05% sodium azide) (24 h), and embedded in optimal cutting temperature (OCT) compound (Tissue-Tek, Sakura). Cryosections of 7 µm thickness were collected. For Masson trichome staining, sections were airdried, and exposed to: Papaniculaus 1 (6 min), running tap water (8 min), 1.2% picric acid (5 min), tap water (1 min), 1% Biebrick scarlet (10 min), 1% phosphor wolfram acid (10 min), 2.5% methyl blue (2 min), acetic acid (30 s), 1% phosphor wolfram acid (5 min), and acetic acid (3 min). Slides were then dehydrated and coverslips were mounted using Aquatex (Sigma Aldrich). For Acta2-staining of mouse aortas, sections were airdried, washed in deionized water (1 min) and PBS (3 min), blocked in 5% normal goat serum (NGS) (ab7481, Abcam) in PBS (30 min), incubated with rabbit anti-mouse Acta2 antibody (ab5694, Abcam) (1:300 in 5% NGS in PBS) (2 h), washed in PBS (3 × 3 min), incubated with Alexa Fluor 555-conjugated goat anti-rabbit IgG (A21428, Invitrogen) (1:300 in 5% NGS in PBS) (1 h), and washed in PBS (3 × 3 min). Coverslips were then mounted with SlowFade Gold Antifade Mountant with DAPI (S36942, Thermo Fisher Scientific).

Human AAA tissue was harvested from the anterior part at open AAA repair procedures from 5 patients (age: 75 ± 5 (mean ± SD) years; 4 males and 1 female) and aortic punches were collected from the ascending aorta of 5 patients undergoing CABG (age: 63 ± 9 (mean ± SD) years, all males). Samples were immersion-fixed in 4% formaldehyde in PBS for 24 h and embedded in paraffin. Masson trichome staining was performed as described for mouse tissue. COL4 and MYH11 immunohistochemistry was performed at the Department of Pathology, Odense University Hospital, using primary antibodies CIV22 (760–2632, Roche, prediluted by Roche) and SMMS-1 (M3558, Dako, 1:100), respectively, utilizing the full-automated OptiView DAB IHC Detection Kit (760–700). Staining protocols conformed with standard practice of the Department of Pathology, Odense University Hospital, including negative controls (staining with indifferent antibodies and omission of primary antibodies) as well as staining of control multiblocks with panels of human tissues.

All donors had given written consent, and the study was performed in accordance with protocols approved by the local ethics committee (S-20100044) and conformed to the principles of the Declaration of Helsinki.

### Plasma samples from the VIVA screening trial

Baseline heparin plasma samples from a randomized population-based screening trial for AAA, PAD, and hypertension in > 50,000 men, 65–74 years of age, in the Mid Region of Denmark^19^ were taken consecutively from 476 AAA patients at the time of diagnosis and from 200 age-matched controls (without AAA or PAD) (all 676 samples were incuded in this study). AAA was defined as a maximal abdominal aortic diameter ≥ 30 mm, and PAD was defined as an ankle–brachial index (ABI) lower than 0.90 or > 1.4. AAA cases among first-degree relatives, smoking status, coexisting diabetes mellitus, hypertension, and use of β-blockers, angiotensin-converting enzyme (ACE) inhibitors, and statins were recorded, as were body mass index (BMI), diastolic- and systolic blood pressure. Maximal anterior–posterior diameter of the infrarenal aorta was measured in the peak of the systole between inner edges of the aorta. The interobserver variation of aortic diameter measurements was 1.52 mm^20^. Growth rates of small AAA in patients kept under surveillance were calculated by individual linear regression analysis, using all observations. Blood samples were centrifuged at 3000×*g* for 12 min, aliquoted, and stored at – 80 °C until analysis. Informed consent was obtained from all subjects before participation, and the study was approved by the Local Ethics Committee of the Mid Region of Denmark, and the data protection agency, and conformed to the principles of the Declaration of Helsinki.

### ELISA measurements of PRO-C4 and C4M

Markers for COL4A1/A2 formation (PRO-C4)^21^ and MMP-2/9-mediated degradation (C4M)^22^ were assessed by ELISA developed by Nordic Bioscience, Herlev, Denmark. Briefly, 96-well streptavidin coated plates were coated with the appropriate biotinylated synthetic peptides and incubated for 30 min at 20 °C. The plates were washed five times with washing buffer (20 mmol/L Tris, 50 mmol/L NaCl, Ph 7.2). Twenty µL standard peptide or pre-diluted heparin plasma sample was added to appropriate wells, followed by incubation with peroxidase-conjugated specific monoclonal antibodies for 1 h at 20 °C. Finally, the plates were washed five times with washing buffer and tetramethylbenzidine (TMB) (cat.438OH, Kem-En-Tec Diagnostics, Taastrup, Denmark) was added, and the plates were incubated for 15 min at 20 °C in darkness. The TMB reaction was stopped by adding 0.18 M H_2_SO_4_ and measured at 450 nm with 650 nm as reference and concentrations read from a calibration curve. All samples were measured within the detection range of the assays.

### Analysis of GWAS data

The GWAS metadata analyzed in this study have been described elsewhere^23^. In brief, a meta-analysis of six independent cohorts, combining 4972 AAA cases and 99,858 controls, was conducted using the METAL software package. Each cohort underwent imputation which was filtered by quality score (Q > 0.9) and only SNPs with minor allele frequencies > 0.05 were included in the final analysis, which consisted of 5,363,770 SNPs assessed in all participants. In the present study, we tested 1667 SNPs in the 557 kb *COL4A1/A2* locus (here defined as the *COL4A1* and *COL4A2* genes (357 kb) flanked by 100 kb border regions) for association with AAA.

### Statistical analyses

Two-group comparisons were performed by Student’s t-tests and presented as mean ± standard error of the mean (SEM). Comparison of two-group XY graphs were performed by two-way ANOVA and presented as mean ± SEM. Explorative proteomics data were analyzed by Student’s t-test for each protein and subsequent false discovery rate (*fdr*) correction for multiple testing, and GO enrichment analysis was performed using default settings of the DAVID Bioinformatics Resources^24,25^. Replicas (*n*) are specified on figures or in figure legends whenever data are not plotted as individual data points. A fixed value of *p* ≤ 0.05 was the criterion for reliable differences between groups, and the significance level symbols used in the figures are: *p ≤ 0.05, **p ≤ 0.01, ***p ≤ 0.001, ****p ≤ 0.0001, while non-significant comparisons are shown as “ns”.

AAA GWAS metadata was analyzed using the METAL software package^26^ and was weighted using the effective sample size [N_eff_ = 4/(1/N_cases_ + 1/N_controls_)] for each cohort.

Statistical analysis of C4M- and PRO-C4-levels in heparin plasma samples from the VIVA screening trial: Dichotomous variables were expressed as proportions and compared by chi-square tests. One sample Kolmogorov–Smirnov tests and normal probability plots (not shown) were used to determine whether continuous variables were normally distributed, and compared between dichotomous variables by 2-tailed unpaired Student’s t-tests and Pearson’s correlation analyses regarding other continuous variables. Baseline variables associated with AAA and COL4A1/A2 fragments with both *p*-values < 10% were considered potential confounders. The COL4A1/A2 fragments were tested as independent predictors of AAA by logistic regression analysis, without and with adjusting for the identified potential confounders: Smoking, hypertension, previous ischemic event in the brain, heart or legs, use of statins, and diastolic blood pressure. The associations between COL4A1/A2 fragments were correlated to maximal aortic diameter and AAA growth rate by univariate and partial Pearson’s correlation analysis without and with adjusting for the aforementioned potential AAA confounders, respectively, supplemented with baseline AAA diameter regarding growth rate. Finally, the biomarkers were tested for independent association with need for surgical evaluation and death of any cause by Cox regression analyses without and with adjusting for the aforementioned potential AAA confounders, respectively.

## Results

### Col4a1/a2 hemizygosity reduces aorta Col4a1/a2 content but does not affect morphometry of the aorta

In contrast to homozygous Col4a1/a2 knockout (KO) (*Col4a1/a2*^*−/−*^) mice, which do not survive intrauterine development^27^, hemizygous *Col4a1/a2* KO (*Col4a1/a2*^+*/−*^) mice are viable and fertile, but have not previously been characterized. We found Col4a1/a2 mRNA and protein to be reduced by 50% in aortas using quantitative PCR (qPCR) and multiple reaction monitoring (MRM) liquid chromatography-mass spectrometry (LC–MS), respectively (Fig. 1a–e), but histologically assessed aortas were indistinguishable between genotypes (Fig. 1f–g and Supplementary Fig. 1). Moreover, no differences were observed in body weight or relative heart mass (Fig. 1h–i).

### Col4a1/a2 hemizygosity augments experimental AAA development

**Figure 2:**
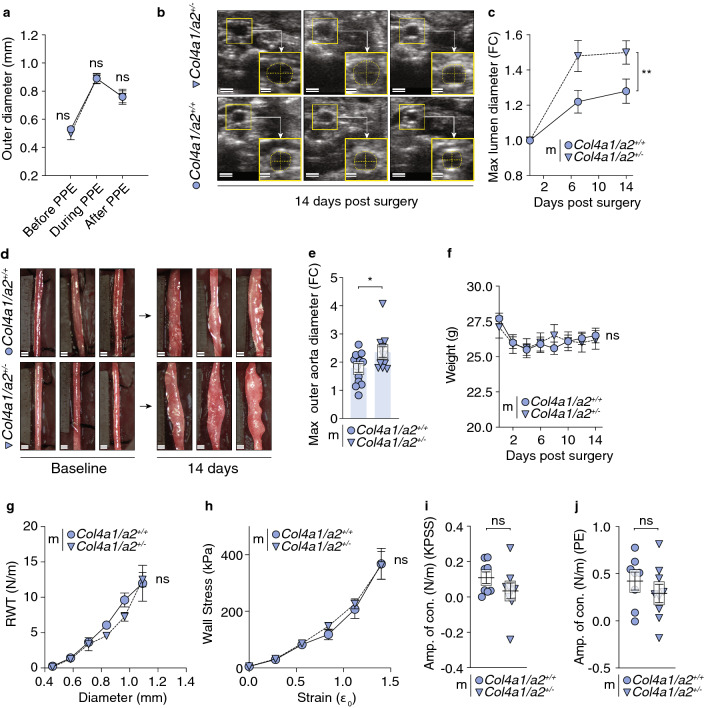
*Col4a1/a2 hemizygosity augments experimental AAA development.* (**a**) Outer aorta diameter before, during and after porcine pancreatic elastase (PPE) surgery as assessed by direct measurement during surgery. (**b**) Representative ultrasound images from six mice (3 *Col4a1/a2*^+*/−*^ vs. 3 *Col4a1/a2*^+*/*+^) 14 days post surgery. Scalebars (overview) = 1 mm; Scalebars (magnification) = 500 µm. (**c**) Maximal lumen diameter measured by ultrasound, expressed as foldchange (FC) relative to baseline diameter. (**d**) Representative images captured from video recordings of aortas from six mice (3 *Col4a1/a2*^+*/−*^ vs. 3 *Col4a1/a2*^+*/*+^) immediately prior to PPE infusion (baseline) and 14 days post surgery. Background has been shaded to assist visualization in print. Scalebars = 0.5 mm. (**e**) Maximal outer aorta diameter measured directly, expressed as FC relative to baseline diameter. For c and e, graphs showing absolute values are presented in Supplementary Fig. 2a,b. (**f**) Weight of *Col4a1/a2*^+*/−*^ mice and *Col4a1/a2*^+*/*+^ littermates before PPE surgery and during the 14 days of AAA development. (**g**, **h**) Raw diameter and resting wall tension measurements from the wire myography normalization procedure (**g**) and the stress strain relationship (**h**) derived from here. (**i**, **j**) Contractile amplitude of the abdominal aorta in response to 80 mM potassium solution (KPSS) (**i**) and 10 µM phenylephrine (PE) (**j**) as assessed by wire myography. Data are presented as mean ± SEM. *p ≤ 0.05, **p ≤ 0.01, ns = non-significant. Unpaired t-test was used in (**a**, **e**, and **i**–**j**). Two-way ANOVA was used in (**c** and **f**–**h**). *n* per group (PPE experiment) = 14–15. *n* per group (wire myography experiment) = 6–9. m = male.

To test for causation between reduced Col4a1/a2 and AAA, we induced AAA in male *Col4a1/a2*^+*/−*^ and *Col4a1/a2*^+*/*+^ mice by the intraluminal porcine pancreatic elastase (PPE) infusion technique^28^. We did not detect differences in outer diameter by direct measurement before, during and after PPE surgery indicating no effect of Col4a1/a2 deficiency on mechanical deformation (Fig. 2a). However, 14 days post-surgery, *Col4a1/a2*^+*/−*^ mice had a modest 17% increase in maximal systolic infrarenal aortic inner (lumen) diameter measured by ultrasonography (Fig. 2b,c and Supplementary Fig. 2a) and a 31% increase in outer aortic diameter relative to diameters before surgery (Fig. 2d,e and Supplementary Fig. 2b). No difference in body weight was observed between groups (Fig. 2f). Our observation that Col4a1/a2 deficiency does not affect biomechanical properties of the aorta was verified in wire myography experiments performed on unchallenged abdominal aortas from an independent mouse cohort. This experiment showed that resting wall tension (Fig. 2g), structural rigidity (Fig. 2h) and β-values (Supplementary Fig. 2c), a measure proportional to Youngs modulus of the arterial wall, were not affected by genotype. Furthermore, the wire myography data indicate that the function of VSMCs under these experimental conditions were not affected by Col4a1/a2 deficiency, since the amplitude of contraction to extracellular high K^+^ (Fig. 2i) or a contractile agonist (Fig. 2j) were similar.

### Col4a1/a2 is required to maintain VSMCs in the contractile phenotype

**Figure 3:**
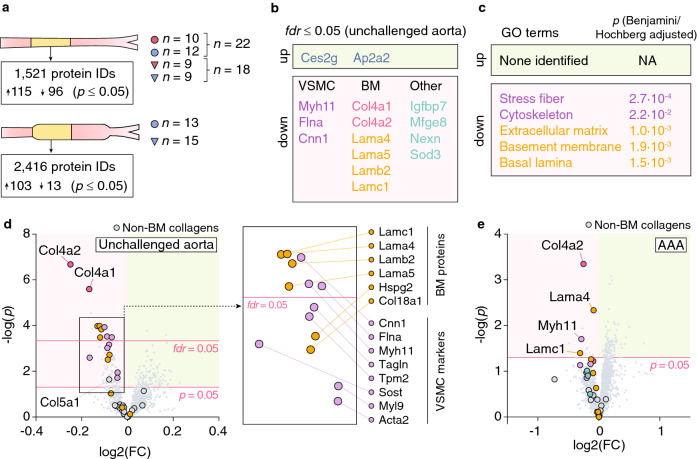
Col4a1/a2 is required to maintain VSMCs in the contractile phenotype. (**a**) Number of proteins identified in unchallenged aortas and proximal halves of AAA lesions, and the number of regulated proteins (uncorrected *p* ≤ 0.05) by the *Col4a1/a2*^+*/−*^ genotype for each condition. For unchallenged aortas, we analyzed 40 samples (aortas from 10 female *Col4a1/a2*^+*/*+^ mice, 12 female *Col4a1/a2*^+*/−*^ mice, 9 male *Col4a1/a2*^+*/*+^ mice, and 9 male *Col4a1/a2*^+*/−*^ mice) mixing sexes to increase statistical power. For AAA lesions, we analyzed 28 samples (AAA from 13 male *Col4a1/a2*^+*/*+^ mice and 15 male *Col4a1/a2*^+*/−*^ mice). (**b**) Significantly regulated proteins in unchallenged aortas after correction for multiple testing (*fdr* ≤ 0.05). No proteins remained significantly regulated for AAA lesions after correction for multiple testing. (**c**) Significantly enriched GO terms among the 94 downregulated proteins (uncorrected *p* ≤ 0.05, excluding Col4a1/a2) in unchallenged *Col4a1/a2*^+*/−*^ aortas. No GO terms were significantly regulated for upregulated proteins (uncorrected *p* ≤ 0.05) in unchallenged aortas, or up- or down-regulated proteins (uncorrected *p* ≤ 0.05, excluding Col4a1/a2) in AAA lesions. (**d**) Volcano plot showing protein level changes in unchallenged *Col4a1/a2*^+*/−*^ aortas relative to *Col4a1/a2*^+*/*+^ aortas. Col4a1/a2 (red), VSMC markers^29^ (magenta), BM proteins (yellow), and non-BM collagens (grey) are magnified for clarity. Identities of regulated (uncorrected *p* ≤ 0.05) VSMC markers and BM proteins are shown in the parallel shifted rectangle. (**e**) Volcano plot showing protein level changes in *Col4a1/a2*^+*/−*^ AAA lesions relative to *Col4a1/a2*^+*/*+^ lesions. The color code of magnified dots is the same as in (**d**). For (**d**, **e**), the red horizontal lines show the corrected (*fdr* = 0.05) and uncorrected (*p* = 0.05) level of significance, respectively. Data in (**b**) and (**d**, **e**) were analyzed by unpaired t-test for each protein followed by *fdr* correction for multiple testing.

To identify effects of Col4a1/a2 deficiency on (1) aortas, which could prime AAA development and/or (2) AAA lesions per se, we analyzed unchallenged aortas and AAA lesions from *Col4a1/a2*^+*/−*^ and *Col4a1/a2*^+*/*+^ mice by LC–MS/MS. Multiple proteins were affected by the *Col4a1/a2*^+*/−*^ genotype (uncorrected *p* ≤ 0.05) in both specimen types (Fig. 3a), but only proteins in unchallenged aortas passed *fdr*-correction for multiple testing (Fig. 3b). Seven of the 11 downregulated proteins in unchallenged aortas (excluding Col4a1/a2) could be categorized as VSMC differentiation markers (Myh11, Flna and Cnn1^29^) or BM components (Lama4, Lama5, Lamb2, Lamc1). Gene Ontology (GO) enrichment analysis of the 94 reduced proteins (uncorrected *p* ≤ 0.05, excluding Col4a1/a2) in unchallenged aortas showed significant enrichment of exactly five GO terms, “stress fiber”, “cytoskeleton”, “extracellular matrix”, “basement membrane” and “basal lamina”, further supporting central effects on BM reduction and VSMC dedifferentiation (Fig. 3c). Several other VSMC markers (Sost, Tpm2, Tagln, Myl9 and Acta2^29^) and BM proteins (Hspg2 and Col18a1) were nominally reduced, while non-BM collagens (with the exception of Col5a1) were unaffected (Fig. 3d). Despite a higher variation in the AAA dataset, Myh11, Lama4 and Lamc1 were also reduced in AAA lesions from *Col4a1/a2*^+*/−*^ mice (Fig. 3e).

To translate the observations from unchallenged mouse aortas to non-lesioned human arteries, we analyzed a proteome dataset of human internal mammary arteries (IMAs) (*n* = 30) previously published from our group^5^, and found significant correlations between COL4A1/A2 and BM components as well as VSMC markers (Table 1 and Supplemetary Fig. 3).

**Table 1:** Correlations of COL4A1 and COL4A2 to BM proteins and VSMC markers in human ITAs (graphical representation of correlations are shown in Supplementary Fig. 4).

Protein	COL4A1			COL4A2		
	R^2^	*p*	Slope (95%)	R^2^	*p*	Slope (95%)
**BM**						
HSPG2	0.21	0.0006	0.17 (0.08–0.27)	0.30	< 0.0001	0.31 (0.19–0.44)
LAMA5	0.15	0.0035	0.20 (0.07–0.34)	0.24	< 0.0001	0.39 (0.21–0.57)
LAMB2	0.32	< 0.0001	0.27 (0.16–0.38)	0.35	< 0.0001	0.43 (0.27–0.58)
LAMC1	0.47	< 0.0001	0.41 (0.29–0.53)	0.44	< 0.0001	0.57 (0.40–0.73)
**VSMC**						
CNN1	0.26	< 0.0001	0.47 (0.25–0.70)	0.44	< 0.0001	0.89 (0.62–1.16)
TAGLN	0.27	< 0.0001	0.60 (0.32–0.87)	0.28	< 0.0001	0.89 (0.51–1.26)
MYH11	0.40	< 0.0001	0.37 (0.24–0.49)	0.60	< 0.0001	0.65 (0.51–0.79)
TPM2	0.31	< 0.0001	0.42 (0.23–0.61)	0.38	< 0.0001	0.76 (0.49–1.03)
ACTA2	0.09	0.0372	0.24 (0.01–0.46)	0.22	0.0004	0.53 (0.25–0.81)
MYL9	0.30	< 0.0001	0.42 (0.24–0.60)	0.39	< 0.0001	0.70 (0.47–0.92)
**Other**						
GAPDH	0.03	0.2480	0.11 (-0.08–0.29)	0.04	0.1392	0.19 (-0.06–0.45)

### The human COL4A1/A2 locus harbors alleles with moderate association to AAA risk

True disease-associated genetic risk variants may be overlooked as a consequence of stringent statistical criteria used in GWAS. Instead, we here used a hypothesis-driven approach based on the largest available AAA GWAS metadata^23^ testing only the 1667 single nucleotide polymorphisms (SNPs) in the 557 kb *COL4A1/A2* locus for association with AAA. We identified a 45 kb region of high linkage disequilibrium in *COL4A2* intron 4 harboring multiple SNPs moderately associated with AAA [lead SNP (rs7320498) *p* = 6.5·10^–5^, OR (A-allele) = 0.887 (95% CI 0.838–0.939)] (Supplementary Fig. 4).

### COL4 is degraded during AAA development and areas with sparse COL4 are depleted in MYH11

**Figure 4:**
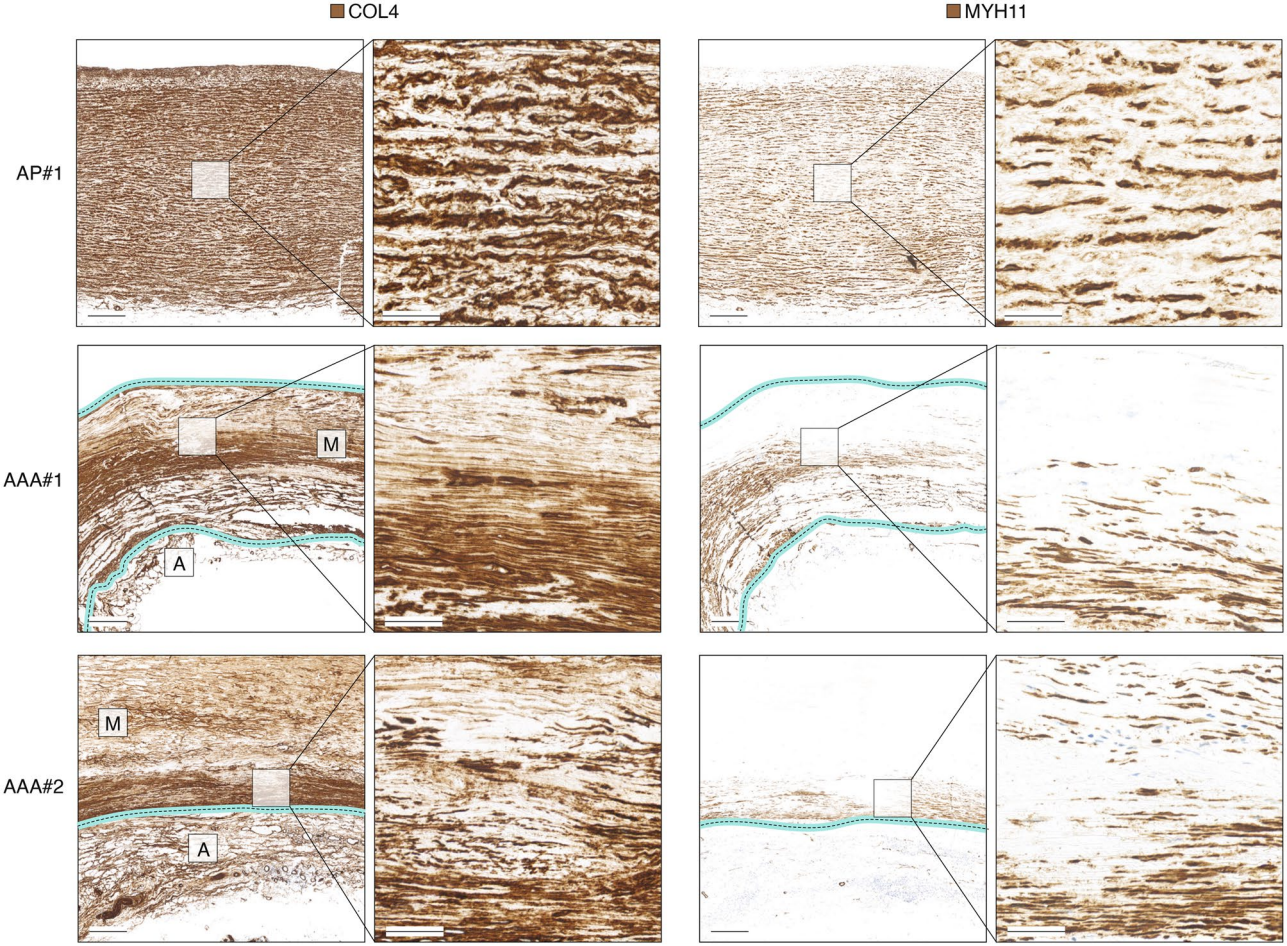
COL4 and MYH11 immunohistochemical staining of non-lesioned ascending aorta and two examples of AAA lesions. To assist assessment of AAA lesions, the partially degraded medial (M) and adventitial (A) layers are labeled on overview images of COL4 stainings, and the media borders are marked with cyan/dotted lines. Images are representative specimens of 5 aortic punch- and 5 AAA-samples analyzed, and images of remaining specimens are shown in Supplementary Fig. 5. Scalebars (overview) = 1 mm; Scalebars (magnification) = 200 µm. AP = Aortic punch (ascending thoracic aorta). #1–#2 refer to sample IDs.

To evaluate whether COL4 could be involved in maintaining VSMC phenotype integrity in human AAA lesions, we assessed human AAA specimens (*n* = 5) obtained from open repair surgery by immunohistochemistry. The density of COL4 staining was reduced, patchy and disorganized as compared to non-lesioned aortas obtained from CABG (*n* = 5), and areas with sparse COL4 had negligible MYH11-staining (Fig. 4 and Supplementary Fig. 5).

### Circulating COL4A1 degradation fragments were independently associated to AAA

Our observations from human AAA specimens suggested that COL4A1/A2 degradation is part of the AAA pathology. To investigate whether degradation of COL4A1/A2 in AAA lesions results in release of circulating COL4A1/A2 fragments in AAA patients, we quantified two well-characterized COL4A1/A2 fragments in plasma samples from a nested case–control cohort of the VIVA screening trial (476 AAA patients and 200 age-matched controls)^19^ (Supplementary Table 1). The MMP-2/9-generated COL4A1 degradation product, C4M, was increased in AAA patients and independently associated with AAA, whereas the COL4A1/A2 formation marker, PRO-C4 was not significantly altered (Table 2). Both markers showed univariate relation to maximal aortic diameters, but only C4M after adjustment for confounding factors (Table 3). Neither marker was related to AAA growth rate or need for surgical repair, whereas both were related to mortality after adjusting for confounders (Supplementary Table 2).

**Table 2:** Univariate and multiple logistic regression analyses of plasma C4M and PRO-C4 levels as independent risk factors of AAA.

	Mean (SD)	*p*	OR	Adjusted OR*
**C4M**				
Controls	13.3 (9.88)	0.004	1.042 (1.015; 1.070) *p* = 0.002	1.090 (1.044; 1.136) *p* ≤ 0.001
AAA	15.5 (9.14)			
**PRO-C4**				
Controls	149.3 (158.6)	0.341	1.001 (0.999; 1.002) *p* = 0.347	1.002 (0.999; 1.004) *p* = 0.123
AAA	158.7 (107.4)			

*Adjusted for Smoking, hypertension, previous ischemic event, statins, and diastolic blood pressure.

**Table 3:** Univariate and partial correlation analyses of the plasma C4M and PRO-C4 levels potential associations with maximal aortic diameter and aneurysmal growth rate.

Pearson’s correlation analysis	Maximal aortic diameter		AAA growth rate	
	Univariate	Partial*	Univariate	Partial**
**C4M**				
r	0.146	0.103	0.029	0.021
*p*	≤ 0.001	0.008	0.585	0.702
**PRO-C4**				
r	0.079	0.036	-0.006	-0.017
*p*	0.037	0.148	0.902	0.758

*Adjusted for smoking, hypertension, previous ischemic event, statins, and diastolic blood pressure.

**Adjusted as above and baseline maximal AAA diameter.

## Discussion

In the present study, we tested the hypothesis that BM proteins COL4A1/A2 have a role in AAA protection.

We used hemizygous *Col4a1/a2*^+*/−*^ mice having a 50% reduction in aorta Col4a1/a2, and found that these mice had increased formation of experimentally-induced AAA. Although homozygous Col4a1/a2 KO (*Col4a1/a2*^*−/−*^) mouse embryos do not survive intrauterine life, it is noteworthy that these embryos show structural deficiency of BM in blood vessels, which appear dilated, and the presence of internal bleedings indicate rupture of blood vessels^27^. These observations support our findings from adult *Col4a1/a2*^+*/−*^ mice.

To address underlying causes of the AAA phenotype, we investigated proteomic consequences of reduced Col4a1/a2 in both unchallenged aortas and AAA lesions. Interestingly, the level of several BM components was reduced in *Col4a1/a2*^+*/−*^ aortas implying that the COL4A1/A2 scaffold is a limiting factor for BM maturation. Indeed, early in vitro studies proposed that COL4A1/A2 is required for the BM assembly process^30,31^, and while it appears that COL4A1/A2 is not required for BM assembly during early embryonic development, it is indispensable for the integrity of the BM at later embryonic stages^27^.

Fully differentiated VSMCs have a high production of BM^32–36^ and VSMCs are assumed to be the principal source of vascular BM. In vitro studies have shown that cultivating VSMCs on BM-rich substrates causes upregulation of myofilaments resulting in a more contractile VSMC phenotype^36–40^. The present study is the first to support these findings in vivo*,* by demonstrating the dependency of VSMC differentiation on an adequate level of BM. We observed a clear reduction in VSMC differentiation markers in *Col4a1/a2*^+*/−*^ aortas in mice, and in human IMAs, COL4A1/A2 correlated with VSMC markers. Importantly, IMAs are musculo-elastic arteries and have different dimensions, properties and embryological origin as compared to abdominal aortas. Therefore, the use of IMAs rather than abdominal aortas is a limitation to this study.

The BM has been proposed to maintain VSMCs in a differentiated state by sustaining an environmental niche and simultaneously providing an adhesive substrate for VSMCs to anchor^6^. Depriving VSMCs from their niche will not only deny them of direct signals from the BM, but concomitantly increase exposure to structural components of the interstitial matrix having a different composition of diffusible factors all affecting VSMC modulation^7^. *Ceteris paribus,* it is conceivable that a shift away from a fully differentiated contractile VSMC phenotype in the normal aorta and throughout disease progression, because of a reduction in COL4A1/A2, could have major implications for AAA development. Phenotypic switching of VSMCs is a central process in AAA pathology^41–43^, and modulated VSMCs secrete both proteolytic enzymes that degrade ECM components and cytokines promoting vascular inflammation^44^, which may accelerate AAA formation. This notion is compatible with mutations of *ACTA2* or *MYH11*, which display severe vascular consequences including *patent ductus arteriosus* and *familial thoracic aortic dissection*^45–52^.

Taken together, it is likely that an inadequate level of COL4A1/A2 renders VSMCs more prone to dedifferentiating at baseline and throughout AAA progression leading to the observed effect of AAA formation. This idea requires further clarification beyond the scope of the present study.

A limitation to our study is that the effect of *Col4a1/a2* hemizygosity in mice may not be confined to the artery wall. This also applies to the modest association signal identified by our hypothesis-driven testing of the *COL4A1/A2* locus. Effects in all tissues comprising epithelia, peripheral nerves, fat- and muscle-cells, which are known to have a COL4A1/A2-containing BM cannot be excluded^53,54^. It is noteworthy, however, that both HANAC patients and mice carrying a missense mutation of Col4a1 first and foremost display a severe vascular phenotype^10,11,55^. This observation points to vascular tissue, as particularly susceptible to dysfunctional COL4A1/A2.

The histological analyses presented in this study show a COL4 distribution in advanced human AAA lesions compatible with increased degradation, in line with a previous report^56^, and furthermore show that MYH11-positive VSMCs primarily remain in areas with dense COL4 staining. Although, the direction of causality (COL4 degradation leading to MYH11 reduction, or VSMC modulation leading to COL4 degradation) or the presence of a common underlying factor cannot be dissected from the simple analyses presented here, these observations are compatible with the notion that COL4A1/A2 reduction may be involved in VSMC dedifferentiation. Indeed, despite variation in the mouse AAA-proteomic data, Myh11 remained nominally reduced suggesting that the effect on VSMCs persists throughout lesion progression. A limitation to the presented histological analysis is that the ascending aorta was used for comparison. The ascending aorta is different from the abdominal aorta in several ways (dimensions, local hemodynamics and embryological origin). Moreover, the histological analyses were based on a pan-COL4 antibody, which does not distinguish COL4A1-COL4A2 from COL4A3-A6. Although this is a limitation to the analysis, unpublished observations from our group show that the COL4A1/A2 chains predominate in vascular tissues, while COL4A3-A6 are scarce if at all measurable.

COL4A1/A2 degradation as a feature of AAA is further supported by our observation that C4M levels are augmented in plasma from AAA-patients. The COL4A1 neoepitope is well-characterized, and known to be formed after MMP-2/9-mediated degradation^57^. Plasma C4M was found to be independent of all relevant risk factors and in addition significantly correlated to maximal abdominal aortic diameter in the VIVA screening trial. This finding is supported by a previous report, where a non-specified Collagen IV epitope was elevated in AAA patients^56^. Interestingly, the COL4A1/A2 formation marker, PRO-C4 was not associated with either AAA-diagnosis or maximal aortic diameter further underlining MMP-2/9-dependent degradation as the principal mechanism resulting in elevated COL4A1 fragment plasma levels. Both tissue and plasma investigations suggest that the artery COL4A1/A2 amount may diminish during development of AAA, an aspect that may further accelerate disease development. Interestingly, several COL4A1/A2 degradation products have been shown to be biologically active. Arresten and Canstatin (degradation products of COL4A1 and -A2, respectively) have anti-angiogenic activity^7^, but whether these peptides play a significant role in AAA pathogenesis remains elusive. Recently, AXT107—a peptide derived from sequences of both COL4A1 and COL4A5^58^ was proposed as a drug displaying strong anti-permeability effects on several vessel types^58,59^—an effect which could also be relevant in the context of AAA^60^.

In conclusion, we provide evidence that reduced baseline artery COL4A1/A2 content increases AAA formation, and that this effect is possibly mediated by mechanisms involving dedifferentiation of VSMCs. Moreover, we show that COL4 degradation is part of the AAA pathology and that COL4A1 degradation fragments are released into the circulation. This may assist future diagnostic and prognostic evaluation of AAA patients.

## Supplementary Information

Supplementary Information.

## Data Availability

The data underlying this article will be shared on reasonable request to the corresponding author.

## Abbreviations

AAA: Abdominal aortic aneurysm
BM: Basement membrane
CABG: Coronary artery bypass grafting
ECM: Extracellular matrix
IMA: Internal mammary artery
MMP: Matrix metalloproteinase
PPE: Porcine pancreatic elastase
VIVA: Viborg vascular (screening trial)

## References

[1] Wanhainen A (2019). Editor’s choice: European Society for Vascular Surgery (ESVS) 2019 clinical practice guidelines on the management of abdominal aorto-iliac artery aneurysms. Eur. J. Vasc. Endovasc..

[2] Solomon CG, Kent KC (2014). Abdominal aortic aneurysms. N. Engl. J. Med..

[3] Lederle FA (2012). The strange relationship between diabetes and abdominal aortic aneurysm. Eur. J. Vasc. Endovasc..

[4] Kristensen KL, Dahl M, Rasmussen LM, Lindholt JS (2017). Glycated hemoglobin is associated with the growth rate of abdominal aortic aneurysms: A substudy from the VIVA (viborg vascular) randomized screening trial. Arterioscleros. Thromb. Vasc. Biol..

[5] Preil SAR (2018). Quantitative proteome analysis reveals increased content of basement membrane proteins in arteries from patients with type 2 diabetes mellitus and lower levels among metformin users. Circ. Cardiovasc. Genet..

[6] Steffensen LB, Rasmussen LM (2018). A role for collagen type IV in cardiovascular disease?. Am. J. Physiol. Hear Circ. Physiol..

[7] Kalluri R (2003). Basement membranes: structure, assembly and role in tumour angiogenesis. Nat. Rev. Cancer.

[8] Schunkert H (2011). Large-scale association analysis identifies 13 new susceptibility loci for coronary artery disease. Nat. Genet..

[9] Program VMV (2019). Genome-wide association study of peripheral artery disease in the Million Veteran Program. Nat. Med..

[10] Plaisier E (2007). COL4A1 mutations and hereditary angiopathy, nephropathy, aneurysms, and muscle cramps. N. Engl. J. Med..

[11] Plaisier E (2010). Novel COL4A1 mutations associated with HANAC syndrome: A role for the triple helical CB3[IV] domain. Am. J. Med. Genet. A.

[12] Itoga NK (2019). Metformin prescription status and abdominal aortic aneurysm disease progression in the U.S. veteran population. J. Vasc. Surg..

[13] Wimmer RA (2019). Human blood vessel organoids as a model of diabetic vasculopathy. Nature.

[14] Fioretto P, Mauer M (2007). Histopathology of diabetic nephropathy. Semin. Nephrol..

[15] Bloksgaard M, Lindsey M, Martinez-Lemus LA (2018). Extracellular matrix in cardiovascular pathophysiology. Am. J. Physiol. Heart. C.

[16] Mulvany MJ, Halpern W (2018). Contractile properties of small arterial resistance vessels in spontaneously hypertensive and normotensive rats. Circ. Res..

[17] Mulorz J (2020). Hyperlipidemia does not affect development of elastase-induced abdominal aortic aneurysm in mice. Atherosclerosis.

[18] Ravnsborg T (2019). First-trimester proteomic profiling identifies novel predictors of gestational diabetes mellitus. PLoS ONE.

[19] Grøndal N, Søgaard R, Henneberg EW, Lindholt JS (2010). The Viborg Vascular (VIVA) screening trial of 65–74 year old men in the central region of Denmark: Study protocol. Trials.

[20] Grøndal N, Bramsen MB, Thomsen MD, Rasmussen CB, Lindholt JS (2012). The cardiac cycle is a major contributor to variability in size measurements of abdominal aortic aneurysms by ultrasound. Eur. J. Vasc. Endovasc..

[21] Leeming DJ (2012). Enzyme-linked immunosorbent serum assay specific for the 7S domain of Collagen Type IV (P4NP 7S): A marker related to the extracellular matrix remodeling during liver fibrogenesis. Hepatol. Res..

[22] Sand JM (2013). MMP mediated degradation of type IV collagen alpha 1 and alpha 3 chains reflects basement membrane remodeling in experimental and clinical fibrosis: validation of two novel biomarker assays. PLoS ONE.

[23] Jones GT (2017). Meta-analysis of genome-wide association studies for abdominal aortic aneurysm identifies four new disease-specific risk loci. Circ. Res..

[24] Huang DW, Sherman BT, Lempicki RA (2009). Systematic and integrative analysis of large gene lists using DAVID bioinformatics resources. Nat. Protoc..

[25] Huang DW, Sherman BT, Lempicki RA (2008). Bioinformatics enrichment tools: Paths toward the comprehensive functional analysis of large gene lists. Nucleic Acids Res..

[26] Recker F, Marquardt K, Redha F, Uhlschmid G, Largiad F (1989). Cyclosporine A impairs wound healing of ureterocystoneostomy in rats: Scanning electron microscopic examination. Urol. Res..

[27] Pöschl E (2004). Collagen IV is essential for basement membrane stability but dispensable for initiation of its assembly during early development. Development.

[28] Pyo R (2000). Targeted gene disruption of matrix metalloproteinase-9 (gelatinase B) suppresses development of experimental abdominal aortic aneurysms. J. Clin. Invest..

[29] Wirka RC (2019). Atheroprotective roles of smooth muscle cell phenotypic modulation and the TCF21 disease gene as revealed by single-cell analysis. Nat. Med..

[30] Timpl R, Wiedemann H, Delden V, Furthmayr H, Kuhn K (1981). A network model for the organization of type IV collagen molecules in basement membranes. Eur. J. Biochem..

[31] Yurchenco PD, Furthmayr H (1984). Self-assembly of basement membrane collagen. Biochemistry.

[32] Hedin U, Bottger BA, Forsberg E, Johansson S, Thyberg J (1988). Diverse effects of fibronectin and laminin on phenotypic properties of cultured arterial smooth muscle cells. J. Cell Biol..

[33] Hedin U, Roy J, Tran PK, Lundmark K, Rahman A (1999). Control of smooth muscle cell proliferation–the role of the basement membrane. Thromb. Haemostasis.

[34] Katsuda S, Okada Y, Minamoto T, Nakanishi I (1987). Enhanced synthesis of type IV collagen in cultured arterial smooth muscle cells associated with phenotypic modulation by dimethyl sulfoxide. Cell Biol. Int. Rep..

[35] Okada Y, Katsuda S, Matsui Y, Watanabe H, Nakanishi I (2008). collagen synthesis by cultured arterial smooth muscle cells during spontaneous phenotypic modulation. Pathol. Int..

[36] Thyberg J (1996). International review of cytology. Int. Rev. Cytol..

[37] Hirose M, Kosugi H, Nakazato K, Hayashi T (1999). Restoration to a quiescent and contractile phenotype from a proliferative phenotype of myofibroblast-like human aortic smooth muscle cells by culture on type IV collagen gels. J. Biochem..

[38] Li J (2014). A novel coating of type IV collagen and hyaluronic acid on stent material-titanium for promoting smooth muscle cell contractile phenotype. Mater. Sci. Eng. C.

[39] Zheng B, Duan C, Clemmons DR (1998). The effect of extracellular matrix proteins on porcine smooth muscle cell insulin-like growth factor (IGF) binding protein-5 synthesis and responsiveness to IGF-I. J. Biol. Chem..

[40] Orr AW (2009). Molecular mechanisms of collagen isotype-specific modulation of smooth muscle cell phenotype. Arterioscl. Thromb. Vasc. Biol..

[41] Peng H (2018). VPO1 modulates vascular smooth muscle cell phenotypic switch by activating extracellular signal-regulated kinase 1/2 (ERK 1/2) in abdominal aortic aneurysms. J. Am. Heart Assoc..

[42] Wen H (2020). Human umbilical cord mesenchymal stem cells attenuate abdominal aortic aneurysm progression in sprague-dawley rats: Implication of vascular smooth muscle cell phenotypic modulation. Stem Cells Dev..

[43] Zhang Z (2019). Knockdown of lncRNA PVT1 inhibits vascular smooth muscle cell apoptosis and extracellular matrix disruption in a murine abdominal aortic aneurysm model. Mol. Cells.

[44] Ailawadi G, Eliason JL, Upchurch GR (2003). Current concepts in the pathogenesis of abdominal aortic aneurysm. J. Vasc. Surg..

[45] Zhu L (2006). Mutations in myosin heavy chain 11 cause a syndrome associating thoracic aortic aneurysm/aortic dissection and patent ductus arteriosus. Nat. Genet..

[46] Harakalova M (2012). Incomplete segregation of MYH11 variants with thoracic aortic aneurysms and dissections and patent ductus arteriosus. Eur. J. Hum. Genet..

[47] Imai Y (2015). A deletion mutation in myosin heavy chain 11 causing familial thoracic aortic dissection in two Japanese pedigrees. Int. J. Cardiol..

[48] Pannu H (2007). MYH11 mutations result in a distinct vascular pathology driven by insulin-like growth factor 1 and angiotensin II. Hum. Mol. Genet..

[49] Kathiravel U (2012). High-density oligonucleotide-based resequencing assay for mutations causing syndromic and non-syndromic forms of thoracic aortic aneurysms and dissections. Mol. Cell Probe.

[50] Guo D-C (2007). Mutations in smooth muscle α-actin (ACTA2) lead to thoracic aortic aneurysms and dissections. Nat. Genet..

[51] Renard M (2013). Novel MYH11 and ACTA2 mutations reveal a role for enhanced TGFβ signaling in FTAAD. Int. J. Cardiol..

[52] Takeda N (2015). A deleterious MYH11 mutation causing familial thoracic aortic dissection. Hum. Genome Var..

[53] Paulsson M (1992). Basement membrane proteins: Structure, assembly, and cellular interactions. Crit. Rev. Biochem. Mol..

[54] Schittny JC, Yurchenco PD (1989). Basement membranes: Molecular organization and function in development and disease. Curr. Opin. Cell Biol..

[55] Agtmael TV (2010). Col4a1 mutation in mice causes defects in vascular function and low blood pressure associated with reduced red blood cell volume. Hum. Mol. Genet..

[56] Holsti M (2018). Circulating vascular basement membrane fragments are associated with the diameter of the abdominal aorta and their expression pattern is altered in AAA tissue. Eur. J. Vasc..

[57] Nielsen MJ (2019). Serum markers of type III and IV procollagen processing predict recurrence of fibrosis in liver transplanted patients. Sci. Rep..

[58] Silva RL (2017). Tyrosine kinase blocking collagen IV–derived peptide suppresses ocular neovascularization and vascular leakage. Sci. Transl. Med..

[59] Mirando AC (2019). A collagen IV–derived peptide disrupts α5β1 integrin and potentiates Ang2/Tie2 signaling. JCI Insight.

[60] Behr-Rasmussen C (2018). Abdominal aortic aneurysms growth is associated with high concentrations of plasma proteins in the intraluminal thrombus and diseased arterial tissue. Arterioscl. Thromb. Vasc. Biol..

