# Supplementary material for: Basement membrane collagen IV deficiency promotes abdominal aortic aneurysm formation
Source: Sci Rep. 2021 Jun 18;11:12903. doi: 10.1038/s41598-021-92303-y (PMC8213747; doi:10.1038/s41598-021-92303-y)
Supplement: Supplementary file 1 — Supplementary Information. [file 41598_2021_92303_MOESM1_ESM.pdf]

Supplementary Material

Basement Membrane Collagen IV Deficiency Promotes Abdominal Aortic Aneurysm Formation.

Steffensen LB[1,2,3], Stubbe J[2], Lindholt JS[1,4], Beck HC[3], Overgaard M[3], Bloksgaard M[2], Genovese F[5], Holm Nielsen S[5,6], Tha MLT[1], Bang-Moeller SK[2], Hong Lin MKT[1], Larsen JH[1,2,3], Hansen DR[1,2,3], Jones GT[7], Bown MJ[4,8], Karsdal MA[5], Rasmussen LM[1,3]*

Author affiliations
1. Centre for Individualized Medicine in Artery Diseases, Odense University Hospital, Odense, Denmark
2. Department of Molecular Medicine, University of Southern Denmark, Odense, Denmark
3. Department of Clinical Biochemistry and Pharmacology, Odense University Hospital, Odense, Denmark
4. Department of Cardiothoracic and Vascular Surgery, Odense University Hospital, Odense, Denmark
5. Nordic Bioscience, Herlev, Denmark
6. Biomedicine and Biotechnology, Technical University of Denmark, Lyngby, Denmark
7. University of Otago, Dunedin, Otago, New Zealand
8. Department of Cardiovascular Sciences and the NIHR Leicester Biomedical Research Centre, University of Leicester, Leicester, United Kingdom

Corresponding author
Lars Melholt Rasmussen
Odense University Hospital
J. B. Winsløws Vej 4, DK-5000, Odense C, Denmark
lars.melholt.rasmussen@rsyd.dk
+45-21158116

Running title: Reduced Collagen IV Promotes AAA Formation.

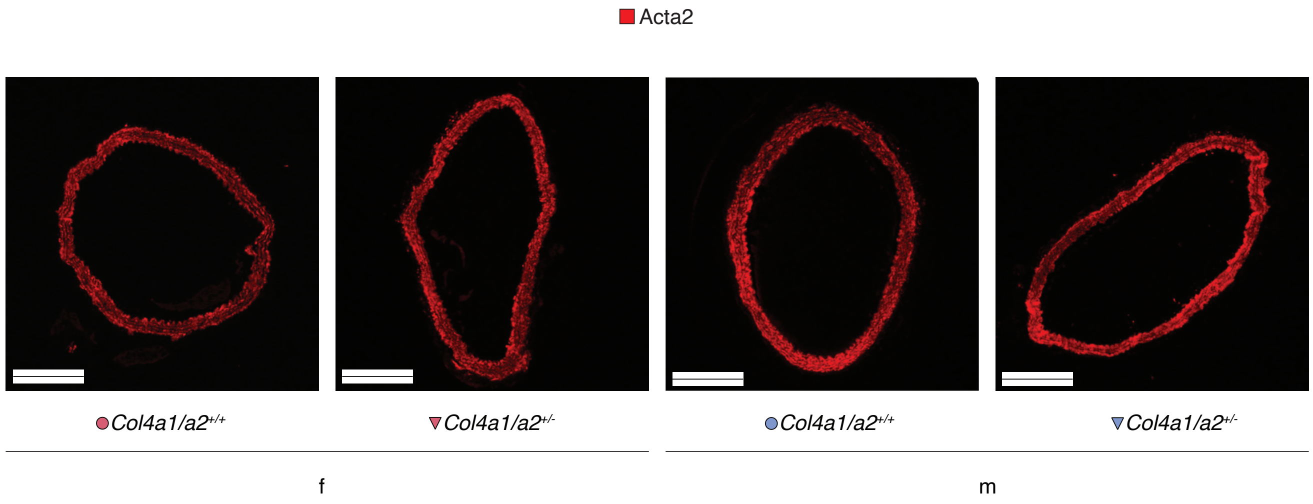

Supplementary Figure 1 | Representative Acta2 immunofluorescent stainings of *Col4a1/a2*$^{+/-}$ and *Col4a1/a2*$^{+/+}$ aortas. Stainings were used to quantify media cross-sectional area (shown in Figure 1g). Scalebars = 100 µm.

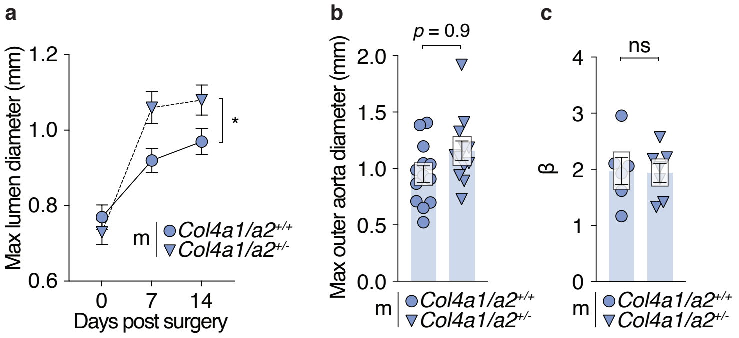

Supplementary Figure 2 | a. Maximal lumen diameter measured by ultrasound in absolute values (as an alternative representation of data shown in Figure 2c). **b.** Maximal outer aorta diameter measured directly using images exemplied in Figure 2d (as an alternative representation of data shown in Figure 2e). **c.** β-value derived from exponential fitting of the stress-strain relationship in Figure 2h. The β-value is proportional to the intrinsic wall stiffness. Data are presented as mean ± SEM. *: $p \leq 0.05$, ns = non-significant. Two-way ANOVA was used in a. Unpaired t-test was used in b-c. *n* per group (PPE experiment) = 14-15. *n* per group (wire myography experiment) = 6-9. m = male.

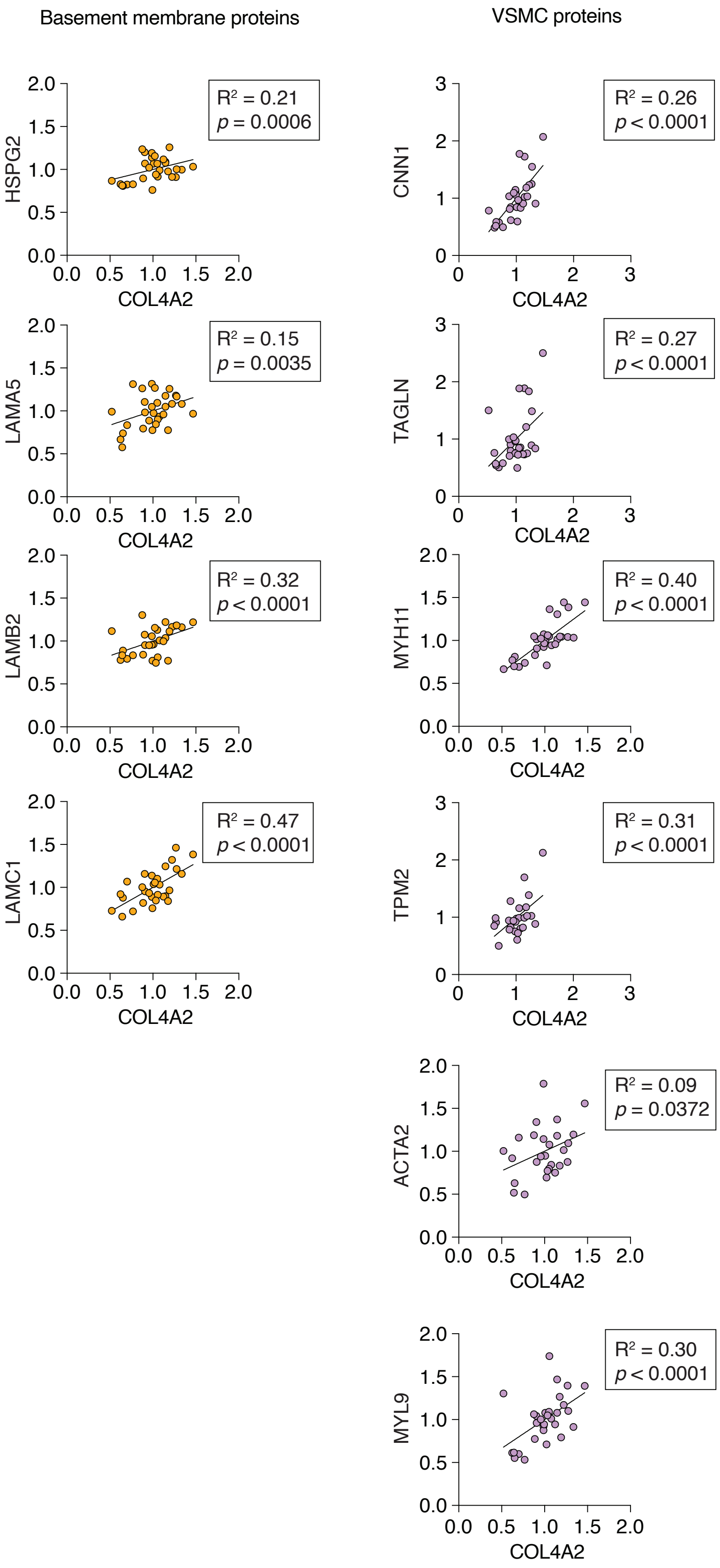

Supplementary Figure 3 | Graphical representation of correlations shown in Table 1. Only correlations between COL4A2 (and not COL4A1) and the panel of proteins are shown for simplicity.

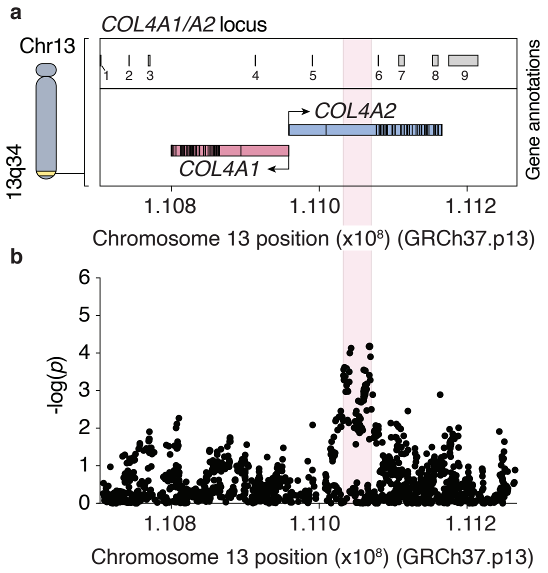

Supplementary Figure 4 | a. The *COL4A1/A2* locus (defined here as the *COL4A1/A2* genes (357 kb) flanked by 100 kb border regions) in chromosomal band 13q34. Besides the *COL4A1/A2* genes, the locus harbors nine genes/pseudogenes (shown in grey): 1. *RP11-403A3-3*, 2. *RN7SL783P*, 3. *RP11-472K17.3*, 4. *RP11-212E4.1*, 5. *MIR8073*, 6. *RP11-291I6.2*, 7. *COL4A2-AS2*, 8. *COL4A2-AS1*, 9. *RAB20*. Exons of *COL4A1/A2* are shown as vertical black lines. **b.** LocusZoom plot of AAA GWAS metadata showing associations between SNPs and AAA in the *COL4A1/A2* locus.

SUPPLEMENTARY FIGURE 5

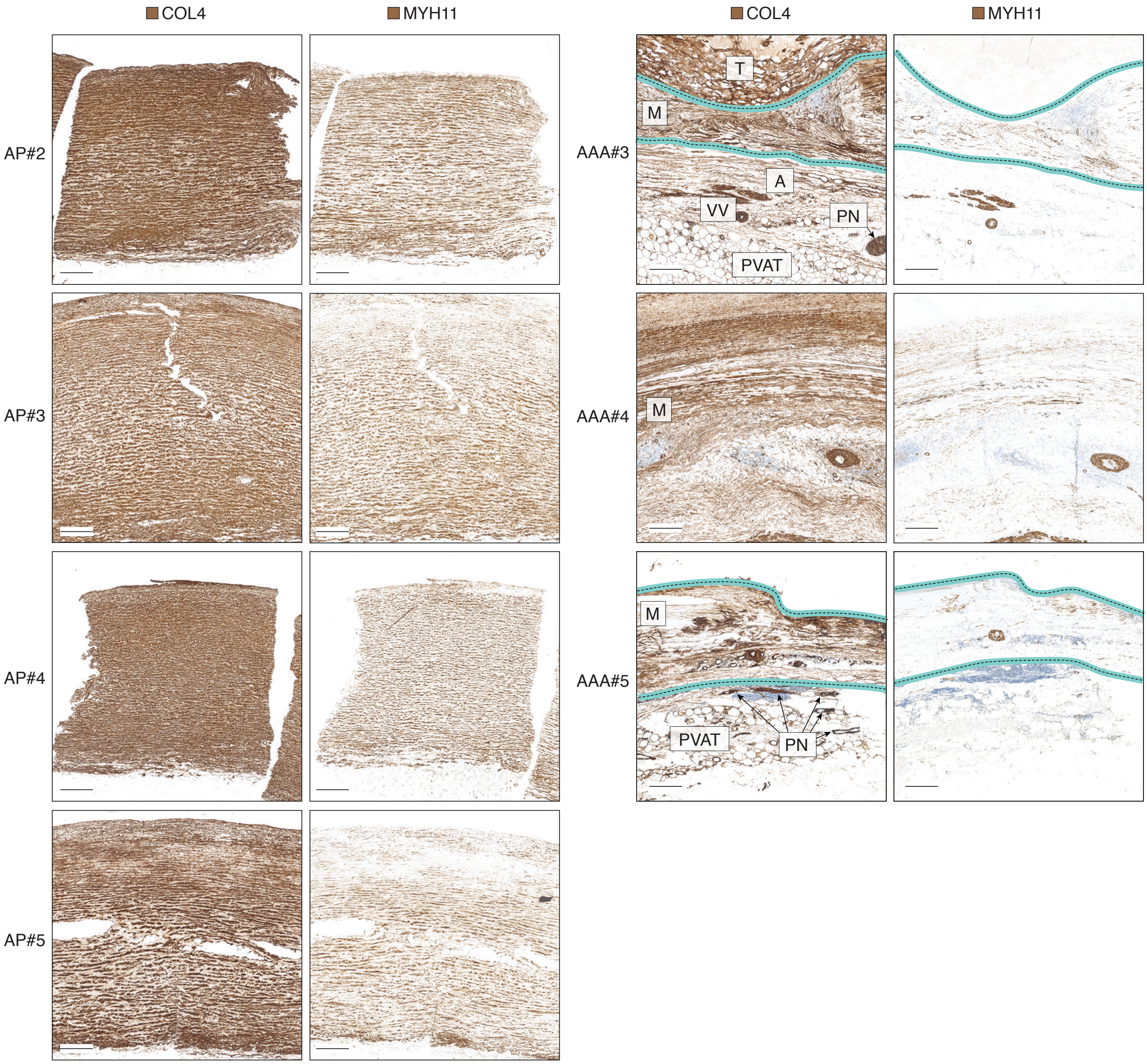

Supplementary Figure 5 | COL4 and MYH11 immunohistochemical staining of four non-lesioned aortas and three of AAA lesions complementing stainings shown in Figure 4. To assist assessment of AAA lesions, the partially degraded medial (M) and adventitial (A) layers are labeled on overview images of COL4 stainings, and the media borders are marked with cyan/dotted lines. In addition, if visible, the mural thrombus (T), vaso vasorum (VV), peripheral nerves (PN), and perivascular adipose tissue (PVAT) are labeled. Scalebars = 1 mm. #2-#5 refer to sample IDs.

SUPPLEMENTARY TABLES

Supplementary Table 1 | Dichotomous and continuous variables between AAA patients and controls and their associations with plasma C4M and PRO-C4 levels.

Dichotomy variables	Control vs. AAA (%)	*p*	C4M (No vs. Yes)	*p*	PRO-C4 (No vs. Yes)	*p*
**Familiar disposition**	3.1<br>7.1	0.037	14.3 (8.1)<br>16.2 (8.51)	0.246	150.3 (99.7)<br>182.8 (213.1)	0.337
**Current smoking**	17.6<br>40.4	≤ 0.001	13.7 (0.74)<br>13.4 (0.68)	≤ 0.001	144.9 (103.9)<br>166.1 (118.4)	0.021
**Diabetes Mellitus**	14.9<br>11.2	0.225	14.7 (8.54)<br>13.4 (6.50)	0.102	154.1 (112.6)<br>137.4 (83.3)	0.102
**Hypertension**	46.3<br>53.4	0.069	14.1 (9.19)<br>14.9 (7.40)	0.204	143.0 (98.0)<br>160.9 (119.1)	0.031
**Previous stroke**	1.0<br>3.7	0.056	14.5 (8.37)<br>14.6 (5.97)	0.963	52.3 (110.3)<br>145.7 (73.5)	0.701
**Previous AMI**	1.5<br>6.3	0.008	14.8 (8.37)<br>15.3 (7.0)	0.524	152.7 (111.2)<br>140.3 (63.8)	0.300
**Previous/current angina pectoris**	5.4<br>10.0	0.055	14.6 (8.51)<br>13.4 (5.75)	0.128	153.2 (112.6)<br>140.4 (66.0)	0.186
**Previous/current PAD**	0.5<br>1.2	0.386	14.5 (8.32)<br>15.8 (7.47)	0.666	151.7 (109.7)<br>190.1 (67.4)	0.184
**Previous ischemic event**	10.3<br>21.1	0.001	14.7 (8.75)<br>13.8 (5.82)	0.194	154.3 (116.9)<br>141.8 (64.1)	0.099
**Use of bronchodilator**	7.9<br>10.7	0.198	14.5 (8.48)<br>14.3 (6.45)	0.851	151.1 (112.1)<br>155.8 (79.1)	0.662
**Use of ACE-inhibitor**	22.2<br>27.3	0.138	14.2 (8.67)<br>15.4 (7.20)	0.085	150.2 (118.0)<br>156.8 (82.9)	0.417
**Use of calcium-blocker**	17.2<br>23.6	0.075	14.5 (8.69)<br>14.5 (7.00)	0.979	148.6 (103.3)<br>164.1 (132.0)	0.187
**Use of beta-blocker**	22.7<br>29.5	0.057	14.6 (9.07)<br>14.1 (5.64)	0.407	152.5 (120.4)<br>147.8 (71.0)	0.531
**Use of anti-blood platelets**	24.6<br>47.3	≤ 0.001	14.2 (7.36)<br>14.8 (9.53)	0.393	151.4 (112.5)<br>152.4 (105.5)	0.900
**Use of statins**	36.5<br>52.5	≤ 0.001	14.4 (9.75)<br>14.2 (6.36)	0.372	159.6 (132.9)<br>143.5 (76.3)	0.049
**Continuous variables**	**Mean (SD)**	***p***	**C4M (Pearson´s r)**	***p***	**PRO-C4 (Pearson´s r)**	***p***
**Age (Years)**	69.7 (2.9)<br>70.0 (2.8)	0.178	-0.200	0.559	-0.015	0.692
**Body mass index (kg/m²)**	26.2 (3.4)<br>27.2 (3.6)	≤ 0.001	0.053	0.168	0.028	0.458
**Systolic blood pressure (mmHg)**	148 (19.4)<br>155 (21.4)	≤ 0.001	0.057	0.136	0.039	0.314
**Diastolic blood pressure (mmHg)**	81.0 (10.5)<br>88.1 (12.2)	≤ 0.001	0.064	0.095	0.045	0.241
**Max. aortic diameter (mm)**	18.3 (2.8)<br>40.9 (11.9)	≤ 0.001	0.146	≤ 0.001	0.079	0.037
**AAA-growth rate**	-<br>3.06 (2.57)	-	0.029	0.585	-0.006	0.902
**ILT (intraluminal thrombus) (prop. of max area)**	-<br>0.274 (0.315)	-	0.051	0.262	0.052	0.251

Potential confounders associated with a *p* ≤ 0.1 for both AAA and a COL4A1/A2 fragments:
Smoking, hypertension, previous ischemic event, use of statins, and diastolic blood pressure.

Supplementary Table 2 | Univariate and multiple Cox regression analyses of plasma C4M and PRO-C4 levels as independent risk factors of need for AAA repair and death (95% C.I. in parenthesis).

	Need for repair		Death	
	**HR**	**Adjusted HR****	**HR**	**Adjusted HR***
**C4M**	1.009 (0.996; 1.022)<br>*p* = 0.160	1.001 (0.983; 1.020)<br>*p* = 0.907	1.020 (1.007; 1.032)<br>*p* = 0.002	1.018 (1.004; 1.032)<br>*p* = 0.012
**PRO-C4**	1.000 (0.999; 1.001)<br>*p* = 0.609	1.000 (0.998; 1.001)<br>*p* = 0.797	1.002 (1.001; 1.002)<br>*p* = 0.001	1.001 (1.000;1.002)<br>*p* = 0.008

*Adjusted for smoking, hypertension, previous ischemic event, statins, and diastolic blood pressure.
**: Adjusted as above and baseline maximal AAA diameter
